# Extending the I-squared statistic to describe treatment effect heterogeneity in cluster, multi-centre randomized trials and individual patient data meta-analysis

**DOI:** 10.1177/0962280220948550

**Published:** 2020-09-21

**Authors:** Karla Hemming, James P Hughes, Joanne E McKenzie, Andrew B Forbes

**Affiliations:** 1 Institute of Applied Health Research, University of Birmingham, Birmingham, UK; 2 Department of Biostatistics, University of Washington, Seattle, WA, USA; 3 School of Public Health and Preventive Medicine, Monash University, Melbourne, Australia

**Keywords:** Cluster-randomized trials, multi-centre randomized trials, individual patient data meta-analysis, treatment effect heterogeneity, I-squared

## Abstract

Treatment effect heterogeneity is commonly investigated in meta-analyses to identify if treatment effects vary across studies. When conducting an aggregate level data meta-analysis it is common to describe the magnitude of any treatment effect heterogeneity using the I-squared statistic, which is an intuitive and easily understood concept. The effect of a treatment might also vary across clusters in a cluster randomized trial, or across centres in multi-centre randomized trial, and it can be of interest to explore this at the analysis stage. In cross-over trials and other randomized designs, in which clusters or centres are exposed to both treatment and control conditions, this treatment effect heterogeneity can be identified. Here we derive and evaluate a comparable I-squared measure to describe the magnitude of heterogeneity in treatment effects across clusters or centres in randomized trials. We further show how this methodology can be used to estimate treatment effect heterogeneity in an individual patient data meta-analysis.

## 1 Introduction

It is well recognized that the effects of treatments will not be the same across all populations.^1^ This variation of the effectiveness of treatments is referred to as treatment effect heterogeneity. Treatment effect heterogeneity might be expected across different patient characteristics (for example age, severity of disease, etc.) and possibly across different settings (for example different geographies, or different centres).^2^ In any realization of treatment effects across different groups the actual variation observed is a combination of “true” treatment effect variation and variation due to sampling error. If it is of interest to estimate how much the “true” treatment effects vary, various analysis methods allow differentiation of these different components of variation.^2–4^

Treatment effect heterogeneity is commonly investigated in systematic reviews and meta-analyses. Indeed, the term treatment effect heterogeneity might be most commonly associated with meta-analyses. Here, treatment effect heterogeneity across studies is not atypical, again because treatments might work differently across different patient groups or across different settings.^5^ Moreover, variation across studies in estimated treatment effects might also occur due to different ways studies are conducted (for example, it might represent subtle differences in the way the intervention is delivered). In a meta-analysis, using study-level aggregate data (where each study provides an estimated treatment effect and standard error), treatment effect heterogeneity is incorporated by the use of what is known as a random effects meta-analysis. Random effect meta-analyses conceptually include parameters to allow for an interaction between treatment effect and study (to model differences in how treatments work across studies). The I-squared statistic has been advocated as a measure to quantify the amount of treatment effect heterogeneity.^6^ The statistic has several advantages, including its interpretation being independent of the treatment effect metric (i.e. relative risk or odds ratio); and, most importantly, I-squared is intuitive and easily understood.^6^ Increasingly, however, individual-level data from studies are available. In such cases, the meta-analysis can be conducted using a one-stage (fitting a generalized linear mixed model with random effects for study by treatment interactions)^4,7,8^ or two-stage (analysing each individual study to estimate treatment effects and then pooling across studies using a random-effects meta-analysis). The I-squared estimate of treatment effect heterogeneity arises naturally in the two-stage approach but there is as yet no recommended way to describe the extent of treatment effect heterogeneity in the one-stage approach.^9–11^

Treatment effect heterogeneity might also be expected within a randomized trial. In cluster randomized trials, it is common practice to use a generalized linear mixed model for analysis.^12^ Note that the random effects typically included in this mixed model – often referred to as random intercepts – describe the differences between the clusters before treatment.^13^ This is in contrast to a meta-analysis where random effects describe the differences between studies in how they react to the treatment. In parallel cluster trials, whilst it may be anticipated that the treatment effects vary across clusters, the trial design does not allow estimation of this. This is because clusters are either fully exposed or unexposed to the intervention and so effects of clusters are confounded with any differential effects of treatments. However, it is possible to estimate treatment by cluster heterogeneity in cluster trials in which treatment is crossed with cluster. This includes stepped-wedge and cross-over cluster trials.^14,15^ In the generalized linear mixed model setting, this is achieved by the addition of a random cluster by treatment interaction (to model between cluster variability in the treatment effect).^16,17^

In individually randomized trials, interest in treatment effect heterogeneity is usually across a small number of sub-groups, such as whether the treatment works differentially in males and females. Consequently, treatment effect heterogeneity is commonly investigated through sub-group analyses.^18^ Here true variation across groups in treatment effects is formally differentiated from sampling variation using interaction tests.^3^ However, in pragmatic trials, interest might also arise in whether a treatment might work differentially across centres in a multi-centre randomized trial. Variation in treatment effects across centres might plausibly represent differential levels of fidelity or surgeon skill for example (i.e. again subtle variations in the way the intervention is delivered). Furthermore, randomization in multi-centre randomized trials is usually stratified by centre and so consequently analysis should also include a random centre effect (similar to how a random cluster effect is included in a cluster randomized trial).^19^ Thus, the analysis setup is very similar to that of a cluster randomized trial, and again in the generalized linear mixed model setting, modelling centre by treatment effect heterogeneity can be achieved by the addition of a random centre by treatment interaction.

Thus, describing and modelling treatment effect heterogeneity in systematic reviews and aggregate level data meta-analysis is common. Yet, when analysing individual-level data from a systematic review there is as yet no parallel measure to the I-squared statistic. In addition, treatment effect heterogeneity can be of interest in primary studies, such as multi-centre randomized trials and cluster randomized trials. Furthermore, there is a strong parallel between how all these data would be analysed in a generalized linear mixed model setup. Consequently, here we propose intuitive metric, derived from the generalized linear mixed model, for quantifying treatment effect heterogeneity in individual patient data meta-analysis, multi-centre individually randomized trials, and cluster randomized trials, derived from the generalized linear mixed model. We investigate how this metric compares with the conventional I-squared metric in meta-analyses and evaluate the performance of the metric across several scenarios (small vs. large studies; low vs. high heterogeneity, etc.). We then illustrate this metric by applying it to several examples (individual patient data meta-analysis, cluster trial and multi-centre randomized trial). We assume a superiority comparison of two treatments, a continuous outcome, cross-sectional designs and an exchangeable correlation structure.

## 2 Background

In the pooling of treatment effect estimates from multiple studies where aggregate data are available, treatment effect heterogeneity (across studies) is incorporated using a random effects meta-analysis.^6^ In addition, the extent of treatment effect heterogeneity can be described using the I-squared statistic and tested for using a statistical test, known as the Q-statistic test. These are all outlined below.

The Q-statistic is a test-statistic for treatment effect heterogeneity in a meta-analysis, such that

(1) $$Q=\sum_{j=1}^{K}{\hat{\sigma }}_{j}^{-2}{\left({\hat{\theta }}_{j}-\hat{\theta }\right)}^{2}$$

where *j* represents study (*K* in total); ${\hat{\theta }}_{j}$ is the (estimated) treatment effect for study *j*; ${\hat{\sigma }}_{j}^{-2}$ is the precision of the estimated treatment effect for study *j* and $\hat{\theta }$ is the weighted pooled estimate of the treatment effect across all studies. Under a random effects meta-analysis, the pooled effect of the treatment is $\hat{\theta }=\frac{\sum {\hat{\theta }}_{j}w_{j}}{\sum w_{j}}$ where $w_{j}=\frac{1}{{\hat{\sigma }}_{j}^{2}+{\hat{\tau }}^{2}}$ , where ${\hat{\tau }}^{2}$ is the (estimated) variance of the distribution of the $\theta_{j}\prime s$ . So, here $\tau^{2}$ is the (true) between-study variation in treatment effects. The parameter $\tau^{2}$ is usually estimated by what is known as the methods of moments or DerSimonian and Laird approach;^20^ although it is increasingly recognized that residual maximum likelihood (REML) approaches have better performance properties.^21^ Under large samples (i.e. large K) $Q\;∼\;X_{K-1}^{2}$ .

Under circumstances where the variance of treatment effect for study *j* does not vary across studies (i.e. $\sigma_{j}^{2}=\sigma^{2}$ ), the intuitive measure of between-study heterogeneity (*I*^2^) is

(2) $$I^{2}=100\%*\frac{{\hat{\tau }}^{2}}{{\hat{\tau }}^{2}+{\hat{\sigma }}^{2}}$$

That is, I-squared is the ratio of the (estimated) between-study variability of the treatment effect ( ${\hat{\tau }}^{2}$ ) to the sum of the (estimated) between-study variability ( ${\hat{\tau }}^{2}$ ) and the (estimated) within-study variability ( ${\hat{\sigma }}^{2}$ ). At this point we note that whilst this ratio of variances looks a little like an intra-cluster correlation (ICC), it is not, a point to which we return to in section 2.2. When $\sigma_{j}^{2}$ varies across studies it turns out that

(3) $$I^{2}=100\%\times \frac{Q-(K-1)}{Q}$$

The predictive interval is used to represent the region in which it is expected that 95% of true trial-specific treatment estimates will fall

(4) $$\left[\hat{\theta }-t_{\alpha /2,K-1}\times \sqrt{({\hat{\tau }}^{2}+SE(}\hat{\theta }{)}^{2})\text{ to }\hat{\theta }+t_{\alpha /2,K-1}\times \sqrt{({\hat{\tau }}^{2}+SE(}\hat{\theta }{)}^{2})\right]$$

where $t_{\alpha /2,K-1}$ is the critical value of the t-distribution with *K* − 1 degrees of freedom and an area of α/2 in both tails and where $SE{\left(\hat{\theta }\right)}^{2}=\sqrt{1/\sum w_{j}}$ is the estimated standard error of the pooled treatment effect $\hat{\theta }$ .^20,22^ This predictive interval provides estimated bounds in which a treatment effect might fall for a study not included in the meta-analysis. This set of methods is referred to as *the two-stage approach* because the data are analysed firstly at the level of the individual study and then pooled in a second step across studies.^8^ We now explore modelling of treatment effect heterogeneity in meta-analysis with individual data and extend these ideas to cluster randomized trials and multi-centre randomized trials.

### 2.1 Modelling treatment effect heterogeneity in a one-stage individual patient data meta-analysis

Now we consider individual patient data meta-analysis where individual rather than aggregate level data are available. To model treatment effect heterogeneity the following model has been proposed^7^

(5) $$\begin{array}{l} y_{\mathrm{ijs}}=\mu +x_{\mathrm{ijs}}\theta +\alpha {\left(C\right)}_{j}+x_{\mathrm{ijs}}\alpha {\left(CT\right)}_{j}+e_{\mathrm{ijs}}\\ e_{\mathrm{ijs}}\;∼\;N[0,\sigma_{e}^{2}] \end{array}$$

where

$$\left(\begin{array}{c} \alpha {\left(C\right)}_{j}\\ \alpha {\left(CT\right)}_{j} \end{array}\right)∼N\left(\left(\begin{array}{c} 0\\ 0 \end{array}\right),\;\left(\begin{array}{cc} \tau_{C}^{2} & \sigma_{CT}\\ \sigma_{CT} & \tau_{CT}^{2} \end{array}\right)\right)$$

and where *y_ijs_* is the outcome for individual *i* ( $i=1\ldots m_{j}$ ), in study *j* ( j=1… K), and in arm *s* (*s *=* *0, 1); *μ* is the mean in the control arm (across all studies); *x_ijs_* is the individual-level treatment indicator for individual *i* in study *j* and arm *s* (1: treatment; 0: control); *θ* is the treatment effect; $\alpha {\left(C\right)}_{j}$ a random intercept for study *j*, with variance $\tau_{C}^{2}$ ; and $\alpha {\left(CT\right)}_{j}$ is a random interaction between study and treatment (with variance $\tau_{TC}^{2}$ ); and *e_ijs_* a residual error term with variance $\sigma_{e}^{2}$ . We use the notation α(C) to denote the random *study* effect, even though α(S) might have been more intuitive, to retain a consistency in terminology in later models where α(C) denotes a random *cluster* effect. The non-zero covariance term *σ_CT_* importantly allows flexibility in whether there is more variation in the control or treatment arm. Note that here $\tau_{C}^{2}$ describes the variation between studies in the absence of treatment. In an individual patient data meta-analysis, this is commonly substituted by a fixed effect and is sometimes referred to as stratifying by study.^8,23^ Equation (5) could be re-formatted replacing $\alpha {\left(C\right)}_{j}$ with fixed study effects. Using fixed effects for studies draws a parallel between the one-stage approach and that commonly fitted under a two-stage approach, a point to which we return to in the discussion. Further, $\tau_{CT}^{2}$ represents the variation between studies in their response to treatment (and so is akin to $\tau^{2}$ in a meta-analysis). Also note that *K* represents the total number of studies; and the total sample size for each study is $M_{j}=2m_{j}$ . We assume here that within any given study the sample size in treatment and control arms are equal, which is not an unreasonable assumption in most cases.

Based on this model we propose the I-squared statistic from a one-stage approach. As a recap the I-squared statistic is the between-study variability of the treatment effect divided by the sum of the between-study variability and the average within-study variability. Consequently, the proposed measure of treatment heterogeneity is

(6) $$I^{2}=100\%\times \frac{\hat{\tau_{CT}^{2}}}{{\hat{\tau }}_{CT}^{2}+\frac{2{\hat{\sigma }}_{e}^{2}}{\bar{m}}}$$

where $\bar{m}$ is the harmonic mean of the study-arm sizes. Thus I-squared is the ratio of the between-study variability of the treatment effect estimated by the model ( ${\hat{\tau }}_{CT}^{2}$ ) divided by the sum of the between-study variability ( ${\hat{\tau }}_{CT}^{2}$ ) and the average within-study variability (estimated as $\frac{\;}{\frac{2{\hat{\sigma }}_{e}^{2}}{\bar{m}}}$ , see Appendix 1 for full justification). Of note, had fixed study effects been used in equation (5), this formula for I-squared would still hold.

The estimated between-study estimate of variability, ${\hat{\tau }}_{CT}^{2}$ can also be used directly along with the estimated treatment effect (averaged across studies), $\hat{\theta }$ , to describe the extent of treatment effect heterogeneity, by the use of

(7) $$\left[\hat{\theta }-t_{\alpha /2,K-1}\sqrt{({\hat{\tau }}_{TC}^{2}+SE(}\hat{\theta }{)}^{2})\text{ to }\hat{\theta }+t_{\alpha /2,K-1}\sqrt{({\hat{\tau }}_{TC}^{2}+SE(}\hat{\theta }{)}^{2})\right]$$

where $SE{\left(\hat{\theta }\right)}^{2}$ is the standard error of the estimated treatment effect $\hat{\theta }$ estimated from the mixed model. Study-specific estimates of treatment effects can be obtained using the best linear unbiased estimates of the study-specific random treatment effects (illustrated in examples that follow).^24^ This set of methods is referred to as one-stage approach because the data are analysed by a single model and do not include the separate phase of analysing the individual studies.

### 2.2 Modelling treatment effect heterogeneity in cluster randomized trials

We now consider a two arm parallel cluster randomized trial. Here treatment is assigned at the level of the cluster. For completeness we build up analysis models from the conventional model to a model with cluster by treatment interactions. The conventional analysis model for this simple setup is

(8) $$\begin{array}{l} y_{ij}=\mu +x_{j}\theta +\alpha {\left(C\right)}_{j}+e_{ij}\\ \alpha j(C)∼N[0,\tau_{C}^{2}]\\ e_{ij}∼N[0,\sigma_{e}^{2}] \end{array}$$

where *y_ij_* is the outcome for individual *i* ( $i=1\ldots M_{j}$ ) in cluster *j* ( j=1… K); *μ* is the mean in the control arm (across all clusters); *x_j_* is the cluster-level treatment indicator (1: treatment; 0: control); *θ* is the treatment effect; $\alpha {\left(C\right)}_{j}$ a random intercept for cluster *j*, with variance $\tau_{C}^{2}$ ; and *e_ij_* a residual error term with variance $\sigma_{e}^{2}$ . Again, we note that here $\tau_{C}^{2}$ has a different interpretation to $\tau^{2}$ in a meta-analysis, where $\tau^{2}$ usually describes variation in response to treatment, because here $\tau_{C}^{2}$ describes the variation between clusters in the absence of treatment. Also note that *K* now represents the total number of clusters (as opposed to number of studies earlier), although we do not change notation as in what follows there is a one to one correspondence between the number of studies and clusters. The cluster size is represented by *M_j_* (note that *M_j_* represented study-size in the one-stage individual patient data meta-analysis).

In stepped-wedge, cluster cross-over trials and other trials where treatment is crossed with clusters, the studies are conducted over multiple periods.^25,26^ In the stepped-wedge trial, exposure to the intervention is partially confounded with time and so analysis models need to adjust for time effects. In cluster cross-over trials, adjustment for time effects can remove any residual time effects. For the analysis of cross-over trials, equation (8) is usually extended by incorporating fixed effects for each time period

(9) $$\begin{array}{l} y_{\mathrm{ijs}}=\mu +\phi_{s}+x_{js}\theta +\alpha {\left(C\right)}_{j}+e_{\mathrm{ijs}}\\ \alpha_{j}∼N[0,\tau_{C}^{2}]\\ e_{\mathrm{ijs}}∼N[0,\sigma_{e}^{2}] \end{array}$$

where *s* is the time period of measurement; $\phi_{s}$ is a fixed categorical effect for measurements taken in period *s* (with $\phi_{1}=0$ for identifiability); and *y_ijs_* now represents the outcome measured on individual *i* ( $i=1\ldots m_{js}$ ), in cluster *j* ( j=1…K ), at time point *s* ( s=1…S ); and so *x_js_* denotes exposure to treatment in cluster *j* at time point *s*; and *μ* represents the mean of the outcome in the first period under the control condition. Note that we use the notation *s* to represent time, when *t* might be more intuitive again to retain a consistency in notation, and because *arm* (denoted by *s*) has been replaced by *time*. Now *m_js_* represents the number of measurements in each cluster-period such that the total cluster size is $M_{j}=\sum_{s}m_{js}$ . The total sample size across all clusters is therefore $\sum_{j}M_{j}$ .

Models are extended to allow for treatment effect heterogeneity by including an interaction term between treatment effect and cluster^16^

(10) $$\begin{array}{l} y_{\mathrm{ijs}}=\mu +x_{js}\theta +\phi_{s}+\alpha {\left(C\right)}_{j}+x_{js}\alpha {\left(CT\right)}_{j}+e_{\mathrm{ijs}}\\ e_{\mathrm{ijs}}∼N[0,\sigma_{e}^{2}] \end{array}$$

where

$$\left(\begin{array}{c} \alpha {\left(C\right)}_{j}\\ \alpha {\left(CT\right)}_{j} \end{array}\right)∼N\left(\left(\begin{array}{c} 0\\ 0 \end{array}\right),\;\left(\begin{array}{cc} \tau_{C}^{2} & \sigma_{CT}\\ \sigma_{CT} & \tau_{CT}^{2} \end{array}\right)\right)$$

Here $\alpha {\left(C\right)}_{j}$ represents the main cluster effect (a random effect) and $\alpha {\left(CT\right)}_{j}$ represents the interaction between cluster and treatment (again a random effect) again with a non-zero covariance term (*σ_CT_*). Random cluster effects thus allow variation across clusters under control condition; and an additional component of variation across clusters under treatment. It is noted that this analysis model is conceptually identical (other than the inclusion of period effects) to the analysis model for an individual patient data meta-analysis (equation (5)). Therefore, for trials where treatment is crossed with cluster, the proposed I-squared is

(11) $$I^{2}=100\%\times \frac{{\hat{\tau }}_{CT}^{2}}{{\hat{\tau }}_{CT}^{2}+\frac{4{\hat{\sigma }}_{e}^{2}}{S\bar{m}}}$$

where $\bar{m}$ is the harmonic mean of the cluster-period sizes; and so $S\bar{m}$ is the average total cluster size. Thus I-squared is the ratio of the between-cluster variability of the treatment effect estimated by the model ( ${\hat{\tau }}_{CT}^{2}$ ) divided by the sum of the between-cluster variability ( ${\hat{\tau }}_{CT}^{2}$ ) and the average within-cluster variability (approximated as $\frac{4{\hat{\sigma }}_{e}^{2}}{S\bar{m}}$ , see Appendix 1 for derivation). At this point it becomes clear that the construct of an I-squared is quite different to the ICC which is not sample size dependent. We also note that here the within-cluster variance has been estimated assuming each cluster size under the control and intervention conditions are approximately equal (see Appendix 1). In cluster cross-over trials, this is likely to be the case. However, in stepped-wedge and other designs in which there are differential numbers of observations within clusters by treatment condition, this value can be estimated more accurately

(12) $$I^{2}=100\%\times \frac{{\hat{\tau }}_{CT}^{2}}{{\hat{\tau }}_{CT}^{2}+{\hat{\sigma }}_{e}^{2}\sum_{j}\frac{1}{{\bar{m}}_{j}}(\frac{1}{s_{j}}+\frac{1}{\left(S-s_{j}\right)})}$$

where *s_j_* denotes the number of time periods that cluster *j* is observed under the intervention condition (Appendix 1). Again, had fixed study effects been used instead of random cluster effects this formula for I-squared would still hold.

Again, the estimated between-study estimate of variability, ${\hat{\tau }}_{CT}^{2}$ can also be used directly along with the estimated treatment effect (averaged across clusters), $\hat{\theta }$ , to describe the extent of treatment effect heterogeneity, by the use of

(13) $$\left[\hat{\theta }-t_{\alpha /2,K-1}\sqrt{({\hat{\tau }}_{TC}^{2}+SE(}\hat{\theta }{)}^{2})\text{ to }\hat{\theta }+t_{\alpha /2,K-1}\sqrt{({\hat{\tau }}_{TC}^{2}+SE(}\hat{\theta }{)}^{2})\right]$$

Here the choice of degrees of freedom is not immediately clear, but we have opted to retain the same number (*K* − 1) that is typically used in a meta-analysis to describe treatment variation across *K* studies, relying on the analogy between clusters and studies. An alternative might be what is sometimes referred to as the between-within degrees of freedom: S(K−1) . And, again cluster-specific estimates of treatment effects can be obtained using the best linear unbiased estimates of the cluster-specific random effects.^24^

### 2.3 Modelling treatment effect heterogeneity in multi-centre randomized trials

Now we consider a multi-centre individually randomized trial. Where randomization is stratified by centre, then precision of the treatment effect is increased by including a random centre effect.^19^ In addition to a random centre effect we also include a random centre by treatment effect (to model treatment effect heterogeneity) and so propose the following model

(14) $$\begin{array}{l} y_{\mathrm{ijs}}=\mu +x_{\mathrm{ijs}}\theta +\alpha {\left(C\right)}_{j}+x_{\mathrm{ijs}}\alpha {\left(CT\right)}_{j}+e_{\mathrm{ijs}}\\ e_{\mathrm{ijs}}∼N[0,\sigma_{e}^{2}] \end{array}$$

where

$$\left(\begin{array}{c} \alpha {\left(C\right)}_{j}\\ \alpha {\left(CT\right)}_{j} \end{array}\right)∼N\left(\left(\begin{array}{c} 0\\ 0 \end{array}\right),\;\left(\begin{array}{cc} \tau_{C}^{2} & \sigma_{CT}\\ \sigma_{CT} & \tau_{CT}^{2} \end{array}\right)\right)$$

where *y_ijs_* is the outcome for individual *i* ( $i=1\ldots m_{j}$ ), in centre *j* ( j=1… K), and in arm *s* (*s *=* *0, 1); *μ* is the mean in the control arm (across all centres); *x_ijs_* is the individual-level treatment indicator for individual *i* in centre *j* and arm *s* (1: treatment; 0: control); *θ* is the treatment effect; $\alpha {\left(C\right)}_{j}$ is a random intercept for centre *j*, with variance $\tau_{C}^{2}$ ; and $\alpha {\left(CT\right)}_{j}$ is a random interaction between centre and treatment (with variance $\tau_{TC}^{2}$ ); and *e_ijs_* is a residual error term with variance $\sigma_{e}^{2}$ . Note here *s* represents arm and not time period. Note also that here $\tau_{C}^{2}$ describes the variation between centres in the absence of treatment; and $\tau_{CT}^{2}$ represents the variation between centres in their response to treatment (and so is akin to $\tau^{2}$ in a meta-analysis); and that *K* now represents the total number of centres; and the total sample size for each centre is $M_{j}=2m_{j}$ (making the assumption that the number in the two treatment arms is the same within any centre).

Consequently, the proposed measure of treatment heterogeneity is (Appendix 1)

(15) $$I^{2}=100\%\times \frac{{\hat{\tau }}_{CT}^{2}}{{\hat{\tau }}_{CT}^{2}+\frac{2{\hat{\sigma }}_{e}^{2}}{\bar{m}}}$$

where $\bar{m}$ is the harmonic mean of the study-arm sizes. Again, had fixed study effects been used instead of random cluster effects this formula for I-squared would still hold. Similar to above a predictive interval can be estimated from model output

(16) $$\left[\hat{\theta }-t_{\alpha /2,K-1}\sqrt{({\hat{\tau }}_{TC}^{2}+SE(}\hat{\theta }{)}^{2})\text{ to }\hat{\theta }+t_{\alpha /2,K-1}\sqrt{({\hat{\tau }}_{TC}^{2}+SE(}\hat{\theta }{)}^{2})\right]$$

And, again study-specific estimates of treatment effects can be obtained using the best linear unbiased estimates of the study-specific random effects.^24^

### 2.4 Other model extensions

There are aspects of the trial analysis which we have not considered so far. For example, in a cluster-cross over design it is common to include a random interaction between cluster and time period of the measurement. So, extending equation (10)

(17) $$\begin{array}{l} y_{\mathrm{ijs}}=\mu +x_{js}\theta +\phi_{s}+\alpha (C{)}_{j}+x_{js}\alpha (CT{)}_{j}+\alpha (CS{)}_{j}+e_{\mathrm{ijs}}\\ e_{\mathrm{ijs}}∼N[0,\sigma_{e}^{2}]\\ \alpha (CS{)}_{j}∼N[0,\tau_{CS}^{2}] \end{array}$$

where

$$\left(\begin{array}{c} \alpha {\left(C\right)}_{j}\\ \alpha {\left(CT\right)}_{j} \end{array}\right)∼N\left(\left(\begin{array}{c} 0\\ 0 \end{array}\right),\;\left(\begin{array}{cc} \tau_{C}^{2} & \sigma_{CT}\\ \sigma_{CT} & \tau_{CT}^{2} \end{array}\right)\right)$$

Here $\alpha {\left(CS\right)}_{j}$ represents a random interaction between the cluster and time-period of measurement, which might or might not be modelled independent to other random effects. Random cluster effects thus allow variation across clusters under control condition; and an additional component of variation across clusters under treatment; and for this variation to depend on time period of measurement.

Defining the I-squared as the ratio of the between-cluster variability of the treatment effect divided by the sum of the between-cluster variability and the average within-cluster treatment effect variance, then

(18) $$I^{2}=100\%\times \frac{{\hat{\tau }}_{CT}^{2}}{{\hat{\tau }}_{CT}^{2}+\frac{4{\hat{\sigma }}_{e}^{2}}{S\bar{m}}}$$

In individually randomized trials, further covariate adjustments are often made for categorical variables used in any randomization stratification or minimization, and again incorporation of these would not change the proposed I-squared construct, but might well change the estimated value in any given setting.

## 3 Simulation study

We now investigate how the proposed I-squared statistic from a one-stage approach compares with the conventional I-squared statistic based on a two-stage approach for the setting of a meta-analysis. The desirable goal of the proposed I-squared statistic is that the measure should be intuitive; should strongly correlate with the conventional I-squared statistic; and that these properties should hold across a range of scenarios. To this end we compare the two I-squared statistics across multiple sets of simulated individual patient data meta-analyses. We do this across several scenarios (small vs. large studies; low vs. high heterogeneity, etc.). We assess correlation and agreement between the two metrics and also assess bias.

### 3.1 Data generation process and simulation scenarios

Data are simulated to represent individual patient level data from multiple two arm individually randomized trials, to replicate the setting of an individual-level data meta-analysis. To this end, data are generated from a linear mixed model (equation (5)) with a random study effect and a random study by treatment interaction (Table 1). We assume two arm individually randomized trials; with either 10, 50 or 100 trials available in each data-set; with number of observations per study-arm either 10, 50, or 100; no overall treatment effect; total variance fixed to 1; and a range of different variance components ( $\tau_{CT}^{2},\;\tau_{T}^{2}$ and $\sigma_{e}^{2}$ ) as described in Table 1. Fixing these parameters then defines the “true” I-squared for each scenario. We then broadly define these true I-squared’s as either low (true I-squared 5% to 20%), moderate (true I-squared 60% to 75%) or high (true I-squared 80% to 97%) treatment effect heterogeneity (Table 1). The large number of trial scenarios allow us to infer performance under settings where fitting linear mixed models are expected to be un-problematic. Scenarios with a small number of trials allow us to infer performance under more realistic settings but where model performance might be poorer due to small sample issues.^27^ In an additional sensitivity analysis, for scenarios with 10 trials, we considered the impact of varying study sizes. In these scenarios, study sizes (number of observations per study-arm) were generated from a zero-truncated negative binomial distribution with coefficient of variation set to 0.7.

**Table 1.** Summary of scenarios considered in the factorial simulation study (in combination these define I-squared).

Study design parameters (considered in factorial combinations)			
Number of studies	Study size per arm	Number of arms	Treatment effect
*K*	*M*	*S*	*θ*
10	10	2	0
50	50	2	0
100	100	2	0
Variance parameters (considered in combinations as listed)			
Study by treatment	Study	Residual	Total
$\tau_{CT}^{2}$	$\tau_{C}^{2}$	$\sigma_{e}^{2}$	∑
0.2500	0.125	0.6250	1
0.1250	0.125	0.7500	1
0.0125	0.125	0.8500	1
0.00125	0.125	0.8725	1

For each simulated data-set we estimate the one-stage and two-stage I-squared statistics (respectively derived from the one-stage and two-stage analyses). For the two-stage approach study-specific treatment effects are estimated (mean differences) along with standard errors (using *regress* command in Stata). These are then pooled across studies using a random-effects meta-analysis, estimating the between study treatment effect heterogeneity estimated using the Dersimonian and Laird approach implemented using the Stata function *metan*. In a sensitivity analysis, we report a subset of results using the REML approach and implemented using the Stata function *metann* to derive an estimate of I-squared.^21^ For the one-stage approach, a linear mixed model is fitted with a random study effect and a random study by treatment interaction, using REML estimation methods (unstructured covariance with maximum number of iterations set at 50) and implemented using the Stata function *mixed*. We then use equation (6) to estimate I-squared. We simulate 10,000 data-sets per scenario (1000 for scenarios considering the impact of varying cluster sizes and using the REML approach). In a sensitivity analysis, we report a subset of results using the mixed effects approach but with fixed study effects (10,000 simulations).

We evaluate the performance by documenting the correlation and agreement between the two I-squared statistics by number of clusters and cluster sizes for the nine scenarios determined by study size and number of studies. We document how many models fail to converge. We also evaluate absolute bias by comparing the estimated I-squared (for both the one-stage and two-stage approaches) to the true I-squared for that particular scenario.

### 3.2 Results

**Figure 1.**
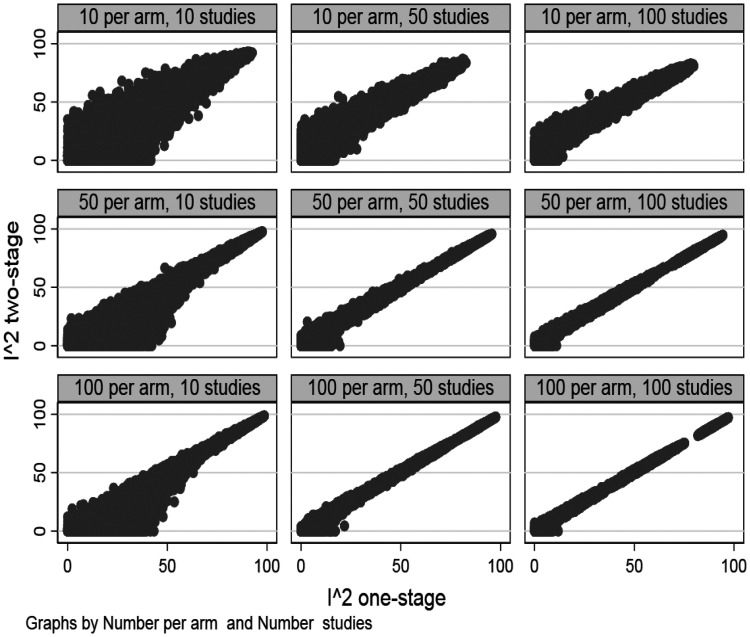
Correlation between I-squared one-stage and I-squared two-stage.

Out of the 360,000 simulated data-sets models failed to converge on 2782 (=0.77%) occasions; and these scenarios are excluded from what follows. For most scenarios all models converged. However, for scenarios with low heterogeneity non-convergence was up to 6% for some scenarios and this was generally higher for scenarios with low heterogeneity (Table S1). Figure 1 and Figure S1 demonstrate a high level of correlation and agreement between the proposed I-squared and the two-stage I-squared, and this is particularly so when there are more than 50 studies and 50 observations per study arm. When there are only 10 studies per arm or only 10 observations per arm, the correlation between the two metrics is lower, especially when there is low heterogeneity (Table S1). In the smallest sample size scenario we looked at, 10 studies and 10 observations per arm, whilst the correlation between the two metrics was lower, there was still a moderate correlation between the two metrics (0.86, Table S1). Agreement between the two metrics was sometimes low, for example when there are either a few studies in the data-set, or many small studies in the data-set, and low heterogeneity (Figure S1). Agreement was higher when there was high heterogeneity, irrespective of the number of studies or size of the studies. The consequences of this low agreement are explored more below by considering the degree of bias in the two metrics.

For most scenarios there was no evidence that either of the I-squared’s were biased by any relevant degree; however, in most scenarios both I-squared measures exhibited some bias. For example, with 100 studies and 50 observations per study arm, and low I-squared, up to about 3% absolute bias was observed (equivalent to 44% on a relative scale) under the two-stage approach and this was similar under the one-stage approach. The magnitudes of these biases look at first hand to be very large, yet when considering how these metrics are interpreted then a difference between for example 7% (truth) and 3% estimated becomes contextually of less importance. Generally the degree of bias mirrored the level of agreement between the two metrics. Bias was generally larger in scenarios where there were only 10 observations per arm (compared with 50 or 100 observations per arm); bias was generally greater for lower compared to higher levels of heterogeneity; and bias tended to be lower where there were more studies. Neither of the two I-squared’s was consistently less biased than the other. When the scenarios were expanded to include varying study-sizes bias under both metrics increased a little and the correlation also decreased a little (Table S2). However, whilst there were some differences in bias between the one-stage and two-stage approach, neither of the one-stage or two-stage approaches clearly stood out as the better performer. Furthermore, in all but the unrealistic scenarios (a meta-analysis of 10 studies of size 10) the correlation between the two metric was above 0.8. Results were similar when using the REML method to estimate between study heterogeneity in the two-stage approach (Table S3). Results were also similar when using fixed instead of random study effects, but with slightly higher levels of correlation between the two metrics (Supplementary Figure S2 and Table S4).

## 4 Examples

We now illustrate these concepts using three examples. The first example is an (simulated) individual patient data meta-analysis and the objective of this example is to illustrate how the proposed I-squared can be estimated from a one-stage approach. In this example, we compare the proposed I-squared with that from a two-stage conventional approach. In the second example, a cluster randomized trial, we illustrate how the I-squared metric can be used to describe treatment effect heterogeneity across clusters. In this example, the intervention has multiple and complex components where it is very conceivable that fidelity of implementation varies across clusters and so effects might vary. In the final example, a multi-centre randomized trial, with randomization stratified by centre, we illustrate how heterogeneity of treatment effects across centres can also be investigated in individually randomized trials. We implement the estimation of these model parameters in Stata using the *mixed* function, using the REML option for estimation of variance parameters.

### 4.1 Example 1: Meta-analysis of simulated individual patient data

**Figure 2.**
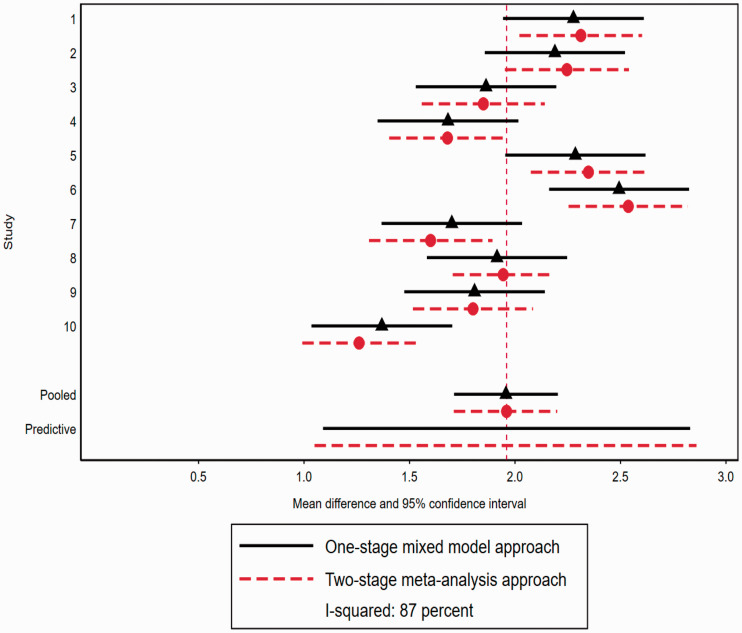
Treatment effect heterogeneity in individual patient data meta-analysis: illustrative example of treatment effect heterogeneity across simulated studies comparing a two-stage random effects meta-analysis with a one-stage mixed model approach (example 1). Red dash lines with circle points represent mean difference and 95% CIs estimated using the two-stage meta-analysis approach and the black line and diamond represent the mean difference and 95% CIs estimated using the one-stage mixed model approach (see text for details). Also presented are predictive intervals for a study not included in meta-analysis.

This example uses simulated individual-level data from 10 parallel two arm individually randomized trials. Consequently the data are available for readers to download and use as an illustrative data-set (https://github.com/karlahemming/One-stage-I-squared). The simulated data have a continuous normally distributed outcome, average treatment effect 2 with residual variance 1 ( $\sigma_{e}^{2}$ ) for 10 studies each with a sample size of 100 per arm, between study variance in absence of treatment 0.1 ( $\tau_{C}^{2}$ ), between study variation in response to treatment 0.2 ( $\tau_{CT}^{2}$ ), corresponding to a ‘true’ I-squared of 91%. The data have then been aggregated to an estimated treatment effect (and variance of the treatment effect) for each study using the *regress* command in Stata (Figure 2, actual numeric values are provided in Supplementary Table S5 for completeness). Data are pooled across studies using a random effects meta-analysis implemented use the Stata function, *metan*, where the between-study variance ( $\tau^{2}$ ) is estimated using the Dersimonian and Laird approach.^28^ Using this approach the I-squared is estimated to be 87.20% (Q-statistic = 70.31 (d.f. = 9); *p* = 0.000; ${\hat{\tau }}^{2}=0.1373$ ). We also estimate I-squared using the REML approach using the Stata function *metaan* (Table S5). The forest plot (Figure 2) illustrates considerable variation across studies in the estimated treatment effects (mean differences). The large I-squared value thus reflects this high degree of heterogeneity. For example, the estimated mean difference for study two is 2.25 (95% CI: 1.95 to 2.54); and for study ten is 1.26 (95% CI: 0.99 to 1.53). The pooled mean difference is 1.96 (95% CI: 1.71 to 2.20); and the corresponding predictive interval, of a treatment effect for a future study, is 1.05 to 2.86.

Whilst in this simulated example, individual-level data are available (since the individual-level data were simulated), this has not been used directly in the estimation of I-squared using the two-stage approach. So, we now illustrate how we propose I-squared can be estimated from a one-stage approach, using the individual-level data directly, fitting model at equation (5) (using the *mixed* command in Stata with REML option) and using equation (6). Using the individual-level data the estimated pooled (across studies) treatment effect is 1.96 (95% CI 1.71 to 2.20); and the estimated between-study variability in treatment effects is ${\hat{\tau }}_{TC}^{2}=0.1360$ ; and the residual variance is $\sigma_{e}^{2}=1.02$ ( $\bar{m}=100$ ), I-squared is estimated to be 86.97%, with prediction interval for a future study, being 1.09 to 2.83.

Using the best linear unbiased predictors we obtain study-specific estimates of treatment effects (Figure 2). For example, the estimated study-specific treatment effect for study two is 2.19 (95% CI: 1.86 to 2.52) and for study ten is 1.37 (95% CI: 1.04 to 1.70). Figure 2 for comparison displays both the estimated study-specific and pooled treatment effects from both the two-stage meta-analysis and one-stage mixed model approach. For this example, there is good agreement between the approaches. We note that results show that the estimated value of the treatment effect heterogeneity random effect (0.14) is lower than the truth (0.2) under both approaches. This likely represents the downward bias in estimation of variance components with few groups.^29^ For illustration we also provide estimates of study-specific treatment effects from a one-stage fixed effects model (Supplementary Figure S3) where the I-squared is estimated to be 87.10%. Appendix 2 provides the Stata code to implement both methods; and the simulated data-set is available as a *csv* file.

### 4.2 Example 2: Cluster randomized stepped-wedge trial

The saving mothers trial is a stepped-wedge cluster randomized trial conducted in Guatemala, where most women have a home-birth and where maternal and neonatal morbidity are high.^30^ This study evaluated an intervention to promote birth in hospital. There were 33 clusters in the study. Clusters were randomized to transition to the intervention at different points in time. Observations were collected monthly for 37 months (i.e. there are 37 time periods in the study). The outcome is the number of births in hospital per month (i.e. a count per cluster-period). For purposes of illustration, we assume the number of births per month is normally distributed and fit a linear mixed model, adjusting for month as a categorical effect.

The treatment effect (mean difference) was estimated to be −0.02 (95% CI: −1.81 to 1.77), so there is no evidence, averaged over clusters, that the treatment has any effect. The estimated variance of the random effect for treatment interaction was ${\hat{\tau }}_{CT}^{2}=15.10$ . The prediction interval is (−8.15 to 8.11) and informs us that for a cluster not included in this study, the estimated cluster-specific treatment effect could be anywhere between an eight count reduction or eight count increase in hospital births per month. The corresponding estimate of I-squared from the one-stage approach is 81.46% (equation (11): $\sigma_{e}^{2}=31.79$ ; $\bar{m}=1$ ; $\tau_{CT}^{2}=15.10$ ; and *S *=* *37); or 67.12% using the approach to correctly acknowledge differential exposure to intervention and control conditions (equation (12)).

**Figure 3.**
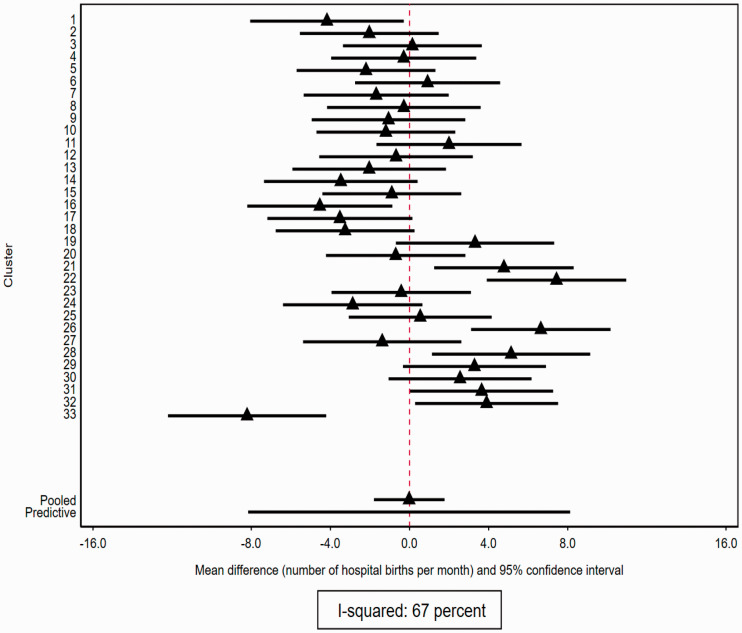
Treatment effect heterogeneity in cluster randomized trials: example includes 33 clusters each with study-specific treatment effect (mean differences) estimated from the best linear unbiased estimators from a one-stage linear mixed model adjusted for time effects with 95% confidence intervals (example 2). Plot also shows estimated average treatment effect across all clusters and predictive interval for a cluster not included in the trial.

We estimate cluster-specific treatment effects using the best linear unbiased predictors (Figure 3). Figure 3 demonstrates considerable variation across clusters in effect sizes. In cluster 33 for example, the intervention is estimated to have a negative effect (reducing the number of hospital births by −8.21 (95% CI −12.21 to −4.21)); whereas in cluster 31 the intervention is estimated to have a positive effect (increasing the number of hospital births by 3.65 (95% CI 0.03 to 7.26)). Note that all of these estimates have been adjusted for time effects.

### 4.3 Example 3: Multi-centre randomized trial

The CHAMPION study is a multi-centre randomized trial comparing two different preventative strategies for postpartum haemorrhage in lower income countries.^31^ The strategies compared were the standard therapy in high income countries, oxytocin that requires cold storage which might not be available in all settings, against an adapted version (heat-stable carbetocin) which does not need cold storage. The study included 23 centres, total sample size of 29,633 with centre size per arm ranging from 84 to 2410. It is plausible that the effectiveness of the treatment might vary by centre as different centres will have differing access to cold storage (which will affect relative comparisons to oxytocin). We consider the continuous outcome of blood-loss after 60 min (natural log-transformed, in millilitres). The trial was designed as a non-inferiority trial although we do not consider this aspect of the trial here. Randomization was stratified by country (there were 10 countries in the study).

We estimate evidence for treatment effect heterogeneity by fitting equation (14) and use equation (15) to estimate the I-squared from a one stage approach. Whilst the study stratified on country and not centre, for the purpose of illustration we fit a model with a random centre effect, and random interaction between centre and treatment. For illustration, we also estimate treatment effects within centres (again using the *regress* command in Stata) and then pool the 23 centre estimates using a random-effects meta-analysis (as an alternative way of estimating treatment effect heterogeneity, see Supplementary Table S6). In what follows we focus on results from the one-stage approach.

**Figure 4.**
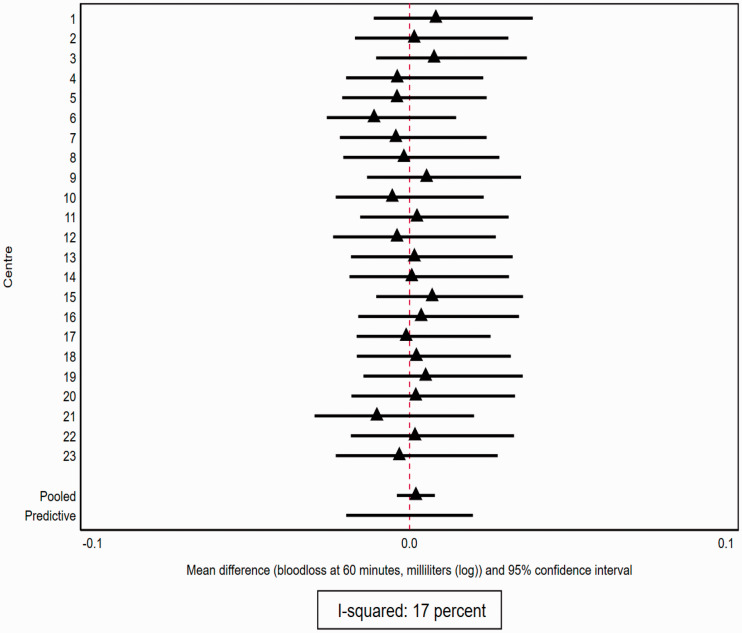
Multi-centre randomized controlled trial: illustrative example of treatment effect heterogeneity across different centres from a one-stage mixed model approach (example 3). Example includes 23 centres each with study-specific treatment effect (mean differences) estimated from the best linear unbiased estimators from a one-stage linear mixed model with 95% confidence intervals. Plot also shows estimated average treatment effect across all centres and predictive interval for a centre not included in the trial.

The treatment effect (mean difference on log scale) was estimated to be 0.01 (95% CI: −0.01 to 0.02), so there is no evidence, averaged over all centres, that there is any difference in effectiveness between the two treatments (on this outcome). The estimated variance of the random effect for treatment interaction was ${\hat{\tau }}_{CT}^{2}=0.0002$ . The prediction interval is (−0.03 to 0.04) and informs us that for a centre not included in this study, the estimated centre-specific treatment effect is likely to be within this fairly narrow range. The corresponding estimate of I-squared from the one-stage approach is 17.16% ( $\sigma_{e}^{2}=0.266$ ; $\bar{m}=617$ ). We estimate centre-specific treatment effects using the best linear unbiased predictors (Figure 4). Figure 4 demonstrates the minimal variation across centres in effect sizes, but considerable uncertainty of effects too.

Of note, the within-centre treatment effects from the mixed models are more precise that those of analysing with within-centre data (for comparison of approaches see Supplementary Table S6). This is a result of the sharing of information in the mixed model (induced by the assumption that the centre-specific random effects come from a common distribution) which leads to a type of shrinkage of the centre-specific estimates.^32^ Hence, the empirical within-study estimates for smaller centres are shrunk further towards the overall mean than are the larger centres. This comes from the random effects assumption that the intervention effects all arise from a common normal distribution, and the small centres are subject to more random variation than larger centres and hence are shrunk further towards the common mean.

## 5 Discussion

Quantifying treatment effect heterogeneity is common in meta-analysis yet uncommon in other settings. In meta-analysis, treatment effect heterogeneity is quantified across studies using the I-squared statistic. Here we have proposed a similar concept to quantify treatment effect heterogeneity in multi-centre and cluster randomized trials and for use in an individual patient data meta-analysis. Correlation is high between the two I-squared statistics, particularly when there is a high degree of heterogeneity, large number of studies and large study sizes. Correlation and agreement is lower when the heterogeneity is low or there are a small number of studies or varying study sizes. We also observed significant degrees of bias in both I-squared metrics, and this was particularly prominent when the number of studies was small and the level of heterogeneity was low. In practice, as long as researchers are mindful of the fact that the absolute value might be some way from the truth, broad interpretation of the metric along the lines of low, medium or high degrees of heterogeneity are likely to be valid. This observed bias is likely to be due to the difficulty in estimation of small variance components in small samples and this would seem to be the case in both the mixed model estimation of the one-stage approach and perhaps under the model estimation of the two-stage approach. It is also worthy of note that in scenarios where there were a few large studies and in scenarios where there were many small studies bias appeared at its greatest. This suggests that the bias we have observed is due to both the estimation of the within-study error term (which is difficult to estimate when each study size is small) and between study treatment variance random effect (which will be difficult to estimate when there are a few studies).

Individual patient data meta-analyses are not as common as aggregate data meta-analyses. In part this is because of the difficulty in obtaining individual-level data, and in part of the added complexity involved. By outlining how to describe treatment heterogeneity in these models, these added complexities will be reduced a little. However, our aim here is not only to develop a methodology for use in individual patient data meta-analyses, but rather capitalize on the similarities of modelling between the field of meta-analysis and the analysis of cluster and multi-centre randomized trials and to promote the investigation of treatment effect heterogeneity in these settings. This will become increasingly important as pragmatic randomized trials become increasingly the norm, and where the evaluation of interventions moves away from regulatory approvals (i.e. does drug A work in a controlled environment) towards facilitating decision making by health care providers (i.e. does intervention X improve outcomes in a real world setting). Evaluations of drugs for regulatory approvals are unlikely to show much variation across centres in efficacy, whereas the evaluation of effectiveness of interventions to inform health care decisions is likely to be context dependent and so some assessment of treatment effect heterogeneity is useful.

The I-squared statistic has several appealing properties, mainly its intuitive interpretation. Facility of interpretation is an important construct. However, the I-squared statistic has some less appealing properties which must also be considered.^33,34^ In particular, whilst the I-squared is not a formal statistical test, it does nonetheless incur the same issues that are problematic in statistical testing. Namely, a low I-squared can indicate no treatment heterogeneity or it can indicate insufficient evidence to make conclusive statements. A high I-squared can indicate clinically important treatment effect heterogeneity or very large sample sizes. This is because the within-cluster component of the I-squared statistic is sample size dependent and goes to zero as the sample size goes to infinity. This therefore means that I-squared goes to 100% as the sample size becomes very large. Quantification of the level of uncertainty in the estimation of I-squared might be of some help. However, this feature is really an issue with the type of construct I-squared is measuring as opposed to a reflection of uncertainty. This serves to highlight the difference between an I-squared measure (which is sample size dependent) and an ICC type measure. Describing treatment effect heterogeneity using $\tau_{CT}^{2}$ is an alternative and is likely to also be considered as an intuitive method by many. Whilst this variance term used without context might not be very meaningful, used in conjunction with a predictive interval it can also create an intuitive measure. Furthermore, estimates of treatment effect heterogeneity from a mixed effects model can be very helpful in designing future studies.^17^

Our proposed estimate for I-squared is based on a mixed effects model where study is modelled as a random effect. In a one-stage approach study is conventionally included as a fixed effect.^8,23^ So, because study effects are included as fixed effects, the estimated treatment effect represents the within-study effect (a conditional effect as opposed to a weighted average of within and between study treatment effects). Although random study effects are sometimes considered in a one-stage approach, for example in multivariate meta-analyses,^35^ this contrasts to a random effects analysis, which estimates the treatment effect as a weighted average of the within-study and between-study treatment effects.^36^ These differences will be inconsequential in analyses in which the distribution of the treatment is the same across studies, as will be common in analyses of randomized trials. By analogy this extends to the analysis of multi-centre stratified trials (here each centre has an equal number of treatment and control observations). However, choice between fixed and random effects will be consequential in the case in cluster cross-over trials where there can be an imbalance of intervention and control observations within clusters and this imbalance differs across clusters (stepped-wedge trials).

There may be other issues to consider when choosing between fixed and random effects. In stratified randomized trials and cluster trials, there are typically (although by no means always) a large number of centres or clusters, whereas individual-level data meta-analyses commonly have only a few studies. In practice, analysis of individual-level data meta-analysis based on random effects models might be at risk of inflated type 1 error rates if they include only a small number of studies.^19^ Therefore in an individual-level data meta-analysis, particularly those based on a small number of studies or non-randomized data (where balance is less likely), a fixed study effect might be more appropriate. However, whilst the proposed methodology drew inspiration from the meta-analysis framework it is not necessarily trying to exactly translate it to this new setting. Moreover, random effect models are conventional in the analysis of cluster trials and are advocated in multi-centre trials,^19^ so we have opted to use random effects. Care is needed however in interpretation of the resulting treatment effects either when using in non-randomized settings or imbalanced designs. Whether or not to include centre (or cluster) as a random or fixed effect thus depends on whether interest is in conditional effects (i.e. fixed effect approach as may be appropriate in a meta-analysis) or in wider population based inferences (i.e. random effects as may be appropriate in CRTs when the clusters are considered as a sample of those from a population of clusters).

There are other reasons to model treatment effect heterogeneity, other than as a way of providing an intuitive description of its extent. Primarily, ignoring treatment effect heterogeneity when it is present can lead to over-precise confidence intervals (i.e. inflated type 1 errors).^37^ We have assumed large sample theory and so require a sufficient number of clusters such that components of variance can be adequately estimated using mixed models. Typically for estimation of fixed effects (for continuous outcomes) this means more than about 40 clusters, centres or studies in total.^38^ Yet, trials and meta-analyses commonly have much fewer than 40 clusters, centres or studies; and furthermore require components of variance (in addition to fixed effects) are well estimated. Small sample corrections are likely to be important in model estimation, but offer limited benefit for estimation of the I-squared parameter as described here, as small sample corrections make adjustments to standard errors of fixed effects and not to the random effects.^39^ Our methodology importantly requires an estimate of the variance of the within-study treatment effect. Where every study is of the same size, this is non-problematic. For studies of varying sizes, we used an approximation based on the harmonic mean. This might be described as a *poor man’s* estimate of the variance. Nonetheless in simulations we found this to have good performance in all but the smallest (and unrealistic) scenarios. However, further work is needed to extend or validate this measure for models which allow for small sample corrections and also for non-continuous outcomes, more extreme forms of varying study sizes (for example a meta-analysis with one very large and many small trials) and in the context of multiple period designs, the impact of time-dependent correlations.^40^

## Supplemental Material

sj-pdf-1-smm-10.1177_0962280220948550 - Supplemental material for Extending the I-squared statistic to describe treatment effect heterogeneity in cluster, multi-centre randomized trials and individual patient data meta-analysis Click here for additional data file. Supplemental material, sj-pdf-1-smm-10.1177_0962280220948550 for Extending the I-squared statistic to describe treatment effect heterogeneity in cluster, multi-centre randomized trials and individual patient data meta-analysis by Karla Hemming, James P Hughes, Joanne E McKenzie and Andrew B Forbes in Statistical Methods in Medical Research

sj-pdf-2-smm-10.1177_0962280220948550 - Supplemental material for Extending the I-squared statistic to describe treatment effect heterogeneity in cluster, multi-centre randomized trials and individual patient data meta-analysis Click here for additional data file. Supplemental material, sj-pdf-2-smm-10.1177_0962280220948550 for Extending the I-squared statistic to describe treatment effect heterogeneity in cluster, multi-centre randomized trials and individual patient data meta-analysis by Karla Hemming, James P Hughes, Joanne E McKenzie and Andrew B Forbes in Statistical Methods in Medical Research

sj-pdf-3-smm-10.1177_0962280220948550 - Supplemental material for Extending the I-squared statistic to describe treatment effect heterogeneity in cluster, multi-centre randomized trials and individual patient data meta-analysis Click here for additional data file. Supplemental material, sj-pdf-3-smm-10.1177_0962280220948550 for Extending the I-squared statistic to describe treatment effect heterogeneity in cluster, multi-centre randomized trials and individual patient data meta-analysis by Karla Hemming, James P Hughes, Joanne E McKenzie and Andrew B Forbes in Statistical Methods in Medical Research

## APPENDIX 1

### A.1 Derivation of I-squared from a one-stage mixed model

#### A.1.1 Individual patient data meta-analysis (or multi-centre randomized trial)

Under the model

(19)
$$\begin{array}{l} y_{\mathrm{ijs}}=\mu +x_{\mathrm{ijs}}\theta +\alpha {\left(C\right)}_{j}+x_{\mathrm{ijs}}\alpha {\left(CT\right)}_{j}+e_{\mathrm{ijs}}\\ e_{\mathrm{ijs}}∼N[0,\sigma_{e}^{2}] \end{array}$$

Recall that *y_ijs_* is the outcome for individual *i* ( $i=1\ldots m_{j}$ ) in study *j* ( j=1…K ) in arm *s* ( s=0,1 ). Furthermore, *μ* is the mean under the control condition; *x_ijs_* is the treatment indicator (1: treatment; 0: control); $\alpha {\left(C\right)}_{j}$ a random intercept for study *j*, with variance $\tau_{C}^{2}$ ; $\alpha {\left(CT\right)}_{j}$ is a random interaction (with variance $\tau_{CT}^{2}$ ) and *e_ijs_* a residual error term with variance $\sigma_{e}^{2}$ . Now *m_j_* represents the number of measurements per arm in each study such that the sample size per study is $M_{j}=2m_{j}$ . The total sample size across all studies is therefore $\sum_{j}M_{j}$ .

The estimated treatment effect for the *jth* study is

$${\hat{\theta }}_{j}={\bar{Y}}_{.j1}-{\bar{Y}}_{.j0}$$

where we use the notation ${\bar{Y}}_{.js}$ to denote averaged over observations within a study and where

$$\begin{array}{l} {\bar{Y}}_{.j1}=\mu +\theta +\alpha (C{)}_{j}+\alpha (CT{)}_{j}+{\bar{e}}_{.j1}\\ {\bar{Y}}_{.j0}=\mu +0+\alpha (C{)}_{j}+0+{\bar{e}}_{.j0} \end{array}$$

so

$${\hat{\theta }}_{j}=\theta +\alpha {\left(CT\right)}_{j}+{\bar{e}}_{.j1}-{\bar{e}}_{.j0}$$

So, the treatment effect for the *jth* study is equal to the true treatment effect (*θ*), plus a random component for the treatment effect varying between studies ( $\alpha {\left(CT\right)}_{j}$ ) and plus the within-study error ( ${\bar{e}}_{.j1}-{\bar{e}}_{.j0}$ ). We can then derive an estimate of the variance of the within-study treatment effect

$$\begin{array}{l} var({\bar{e}}_{.j1}-{\bar{e}}_{.j0})=\mathrm{var}(\frac{\sum_{i=1}^{m_{j}}e_{ij1}}{m_{j}})+\mathrm{var}(\frac{\sum_{i=1}^{m_{j}}e_{ij0}}{m_{j}})\\ var({\bar{e}}_{.j1}-{\bar{e}}_{.j0})=2\frac{\sigma_{e}^{2}}{m_{j}} \end{array}$$

Therefore the I-squared is

(20)
$$I^{2}=\frac{\tau_{CT}^{2}}{\tau_{CT}^{2}+2K\sigma_{e}^{2}\sum_{j}\frac{1}{m_{j}}}=\frac{\tau_{CT}^{2}}{\tau_{CT}^{2}+2\frac{\sigma_{e}^{2}}{m_{¯}}}$$

where $\bar{m}$ is the harmonic mean of the study sizes ( $=K\sum_{j}\frac{1}{m_{j}}$ ). Now, making an assumption that the sample size observed in each study and under control and intervention condition is *m* then

$$\begin{array}{l} var({\bar{e}}_{.j1}-{\bar{e}}_{.j0})=\frac{\sum_{i=1}^{m}var(e_{ij1})}{m^{2}}+\frac{\sum_{i=1}^{m}var(e_{ij2})}{m^{2}}\\ var({\bar{e}}_{.j1}-{\bar{e}}_{.j0})=\frac{2\sigma_{e}^{2}}{m} \end{array}$$

So, averaging across the *K* studies, the I-squared is

(21)
$$I^{2}=\frac{\mathrm{Var}(\alpha (CT))}{\mathrm{Var}(\alpha (CT)+\frac{2Var(e)}{m}}=\frac{\tau_{CT}^{2}}{\tau_{CT}^{2}+\frac{2\sigma_{e}^{2}}{m}}$$

#### A.2.1 Cross-over cluster trials

Under the model

(22)
$$\begin{array}{l} y_{\mathrm{ijs}}=\mu +x_{js}\theta +\alpha (C{)}_{j}+x_{js}\alpha (CT{)}_{j}+\phi_{s}+e_{\mathrm{ijs}}\\ e_{\mathrm{ijs}}∼N[0,\sigma_{e}^{2}] \end{array}$$

Recall that *y_ijs_* is the outcome for individual *i* ( $i=1\ldots m_{js}$ ) in cluster *j* ( j=1…K ) at time point *s* ( s=1…S ). Furthermore, *μ* is the mean under the control condition in the first period; *x_js_* is the treatment indicator (1: treatment; 0: control); $\phi_{s}$ is a fixed categorical effect for measurements taken in period *s* (with $\phi_{1}=0$ for identifiablity); $\alpha {\left(C\right)}_{j}$ is a random intercept for cluster *j*, with variance $\tau_{C}^{2}$ ; $\alpha {\left(CT\right)}_{j}$ is a random interaction (with variance $\tau_{CT}^{2}$ ); and *e_ijs_* is a residual error term with variance $\sigma_{e}^{2}$ . Now *m_js_* represents the number of measurements in each cluster-period such that $M_{j}=\sum_{s}m_{js}$ . The total sample size across all clusters is therefore $\sum_{j}M_{j}$ .

The estimated treatment effect for the *jth* study is

$${\hat{\theta }}_{j}={\bar{Y}}_{.j(x_{js}=1)}-{\bar{Y}}_{.j(x_{js0}=0)}$$

where

$$\begin{array}{l} {\bar{Y}}_{.j(x_{js}=1)}=\mu +\theta +\alpha (C{)}_{j}+\alpha (CT{)}_{j}+{\bar{\phi }}_{.j1}+{\bar{e}}_{.j1}\\ {\bar{Y}}_{.j(x_{js}=0)}=\mu +0+\alpha (C{)}_{j}+0+{\bar{\phi }}_{.j0}+{\bar{e}}_{.j0} \end{array}$$

and where ${\bar{\phi }}_{.j1}$ represents the average (weighted by the number of periods cluster *j* spends in the intervention condition) period effect under the intervention condition (and receptively under the control condition), so

$${\hat{\theta }}_{j}=\theta +\alpha {\left(CT\right)}_{j}+{\bar{\phi }}_{.j1}-{\bar{\phi }}_{.j0}+{\bar{e}}_{.j1}-{\bar{e}}_{.j0}$$

So, the treatment effect for the *jth* cluster is equal to the true treatment effect (*θ*), plus an adjustment for time confounding, plus a random component for the treatment effect varying between clusters ( $\alpha {\left(CT\right)}_{j}$ ) and plus the within-cluster error ( ${\bar{e}}_{.j1}-{\bar{e}}_{.j0}$ ). We ignore period effects in what follows, as these are fixed effects.

Introducing a new notation, *s_j_* to denote number of time periods that cluster *j* is observed under the intervention condition, we can then derive an estimate of the variance of the within-cluster treatment effect

$$\begin{array}{l} var({\bar{e}}_{.j1}-{\bar{e}}_{.j0})=\mathrm{var}\left(\frac{\sum_{s=1}^{s_{j}}\sum_{i=1}^{m_{js}}e_{ij1}}{m_{js}s_{j}}\right)+\mathrm{var}\left(\frac{\sum_{s_{j}+1}^{S}\sum_{i=1}^{m_{js}}e_{ij0}}{m_{js}(S-s_{j})}\right)\\ var({\bar{e}}_{.j1}-{\bar{e}}_{.j0})=\frac{\mathrm{var}\left(\sum_{s=1}^{s_{j}}\sum_{i=1}^{m_{js}}e_{ij1}\right)}{m_{js}^{2}s_{j}^{2}}+\frac{\mathrm{var}\left(\sum_{s_{j}+1}^{S}\sum_{i=1}^{m_{js}}e_{ij0}\right)}{m_{js}^{2}{\left(S-s_{j}\right)}^{2}}\\ var({\bar{e}}_{.j1}-{\bar{e}}_{.j0})=\frac{\sum_{s=1}^{s_{j}}\sum_{i=1}^{m_{js}}\sigma_{e}^{2}}{m_{js}^{2}s_{j}^{2}}+\frac{\sum_{s_{j}+1}^{S}\sum_{i=1}^{m_{js}}\sigma_{e}^{2}}{m_{js}^{2}{\left(S-s_{j}\right)}^{2}} \end{array}$$

Now making the approximation that each cluster-period size is *m_j_*

$$\begin{array}{l} var({\bar{e}}_{.j1}-{\bar{e}}_{.j0})=\frac{m_{j}s_{j}\sigma_{e}^{2}}{m_{j}^{2}s_{j}^{2}}+\frac{m_{j}(S-s_{j})\sigma_{e}^{2}}{m_{j}^{2}{\left(S-s_{j}\right)}^{2}}\\ var({\bar{e}}_{.j1}-{\bar{e}}_{.j0})=\frac{\sigma_{e}^{2}}{m_{j}s_{j}}+\frac{\sigma_{e}^{2}}{m_{j}(S-s_{j})}\\ var({\bar{e}}_{.j1}-{\bar{e}}_{.j0})=\frac{\sigma_{e}^{2}}{m_{j}}\left(\frac{1}{s_{j}}+\frac{1}{\left(S-s_{j}\right)}\right) \end{array}$$

So, averaging across the *K* clusters, the proposed I-squared is

(23)
$$I^{2}=\frac{{\hat{\tau }}_{CT}^{2}}{{\hat{\tau }}_{CT}^{2}+{\hat{\sigma }}_{e}^{2}\sum_{j}\frac{1}{m_{j}}\left(\frac{1}{s_{j}}+\frac{1}{\left(S-s_{j}\right)}\right)}$$

Now, making an approximation and assuming that the average number of periods observed under the control and intervention condition is S/2 then and also that the harmonic mean of the cluster-periods sizes is $\bar{m}$

$$\begin{array}{l} var({\bar{e}}_{.j1}-{\bar{e}}_{.j0})=\frac{\sigma_{e}^{2}}{\bar{m}}\left(\frac{1}{s_{j}}+\frac{1}{\left(S-s_{j}\right)}\right)\\ var({\bar{e}}_{.j1}-{\bar{e}}_{.j0})\approx \frac{\sigma_{e}^{2}}{\bar{m}}\left(\frac{2}{S}+\frac{2}{S}\right)\\ var({\bar{e}}_{.j1}-{\bar{e}}_{.j0})\approx \frac{4\sigma_{e}^{2}}{\bar{m}S} \end{array}$$

So, the proposed I-squared is

(24)
$$I^{2}=\frac{\mathrm{Var}(\alpha (CT))}{\mathrm{Var}(\alpha (CT)+\frac{2Var(e)}{m}}=\frac{\tau_{CT}^{2}}{\tau_{CT}^{2}+\frac{4\sigma_{e}^{2}}{mS}}$$

Note that here the multiplier of *σ_e_* is 4/mS whereas it was 2/m in the meta-analysis setting, and this is explained by how here *S* represents the number of time periods in the cluster cross-over design, and in the parallel setting *S* represents the number of arms; but in both cases the multiplier is 4/SS where *SS* is the sample size in each study or centre. We also note that in the case of cluster-crossover trials, where each cluster spends approximately equal periods in the control and intervention arm that this approximated version of the I-squared should work well. In stepped-wedge trials and other cluster trials, where each cluster spends different numbers of periods exposed to intervention and control conditions, the non-approximated version of the I-squared statistic will be more appropriate. In cluster-cross over trials where cluster sizes vary, the exact approach might also be preferable as this allows for varying cluster sizes.

### APPENDIX 2

#### B.1 Two-stage and one-stage analysis code

****Two-stage approach

import excel “SimulatedIPDEstimates V2.xlsx”, sheet(“IPD)” f irstrow

statsby _b _se, by(study) clear: regress y trt

set scheme s1mono

metan _b_trt _se_trt, random texts(120) nowt effect(mean difference) force xlabel(0.9, 3) nulloff rfdist

metaan _b_trt _se_trt, reml//using REML//

****One-stage approach

*Fit mixed models

clear

import excel “SimulatedIPDEstimates V2.xlsx”, sheet(“IPD”) f irstrow

*noi noi mixed y i.trt i.study ǁ study: trt, var reml matlog iterate(10)//Fixed effects//

noi mixed y i.trt ǁ study: trt, var reml matlog iterate(10)

*Extract variance components

mat b = e(b)

local sig2CT = exp(2* _b[lns1_1_1: _cons])

local sig2E = exp(2*_b[lnsig_e: _cons])

*Estimate average study-arm size

means n_study_arm if trt==1//Harmonic mean study size of each treatment arm

local n_study_hm = r(mean_h)

*Derive I-squared

local within_hm = ‘sig2E'*(2/‘n_study_hm')

local I2_est_hm = 100*‘sig2CT'/(‘sig2CT' + ‘within_hm')

*Display estimated I-squared

di ‘I2_est_hm'

*Predictive interval

quietly: tab study

local n_studies=r(r)//degrees of freedom

di ‘n_studies'

local critical_t=invttail(‘n_studies',0.025)

local seb=_se[1.trt]

local LCI_t=_b[1.trt]-‘critical_t'*sqrt(‘sig2CT'+‘seb'^2)

di ‘LCI_t'

local UCI_t=_b[1.trt]+‘critical_t'*sqrt(`sig2CT'+‘seb'^2)

di ‘UCI_t'

*Best Linear Unbiased Predictors

predict r1 r0, reffects

gen t1=_b[1.trt]+r1

predict r1_sd r0_sd, reses

gen t1_lci=t1-1.96*r1_sd

gen t1_uci=t1 + 1.96*r1_sd

*Create plot

sort study

drop if trt==0

collapse(mean) t1 r1 r0 r1_sd r0_sd t1_lci t1_uci, by(study)

sort study

metan t1 t1_lci t1_uci, fixed favours (favours control # favours treatment) nowt nooverall effect(mean difference)

set scheme s1mono
