# Supplementary figures and images for: Extending the I-squared statistic to describe treatment effect heterogeneity in cluster, multi-centre randomized trials and individual patient data meta-analysis
Source: Stat Methods Med Res. 2020 Sep 21;30(2):376–95. doi: 10.1177/0962280220948550 (PMC8173367; doi:10.1177/0962280220948550)

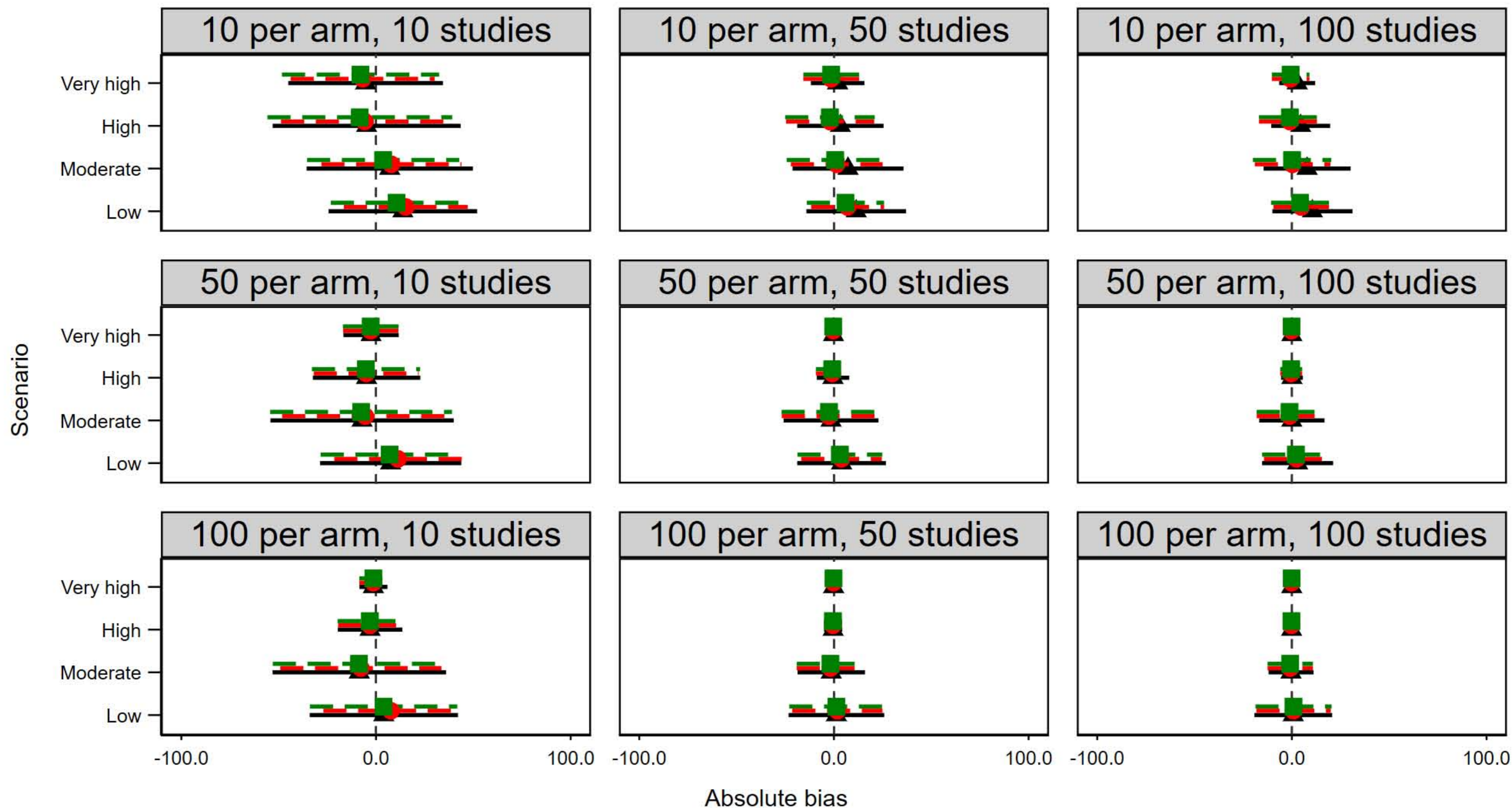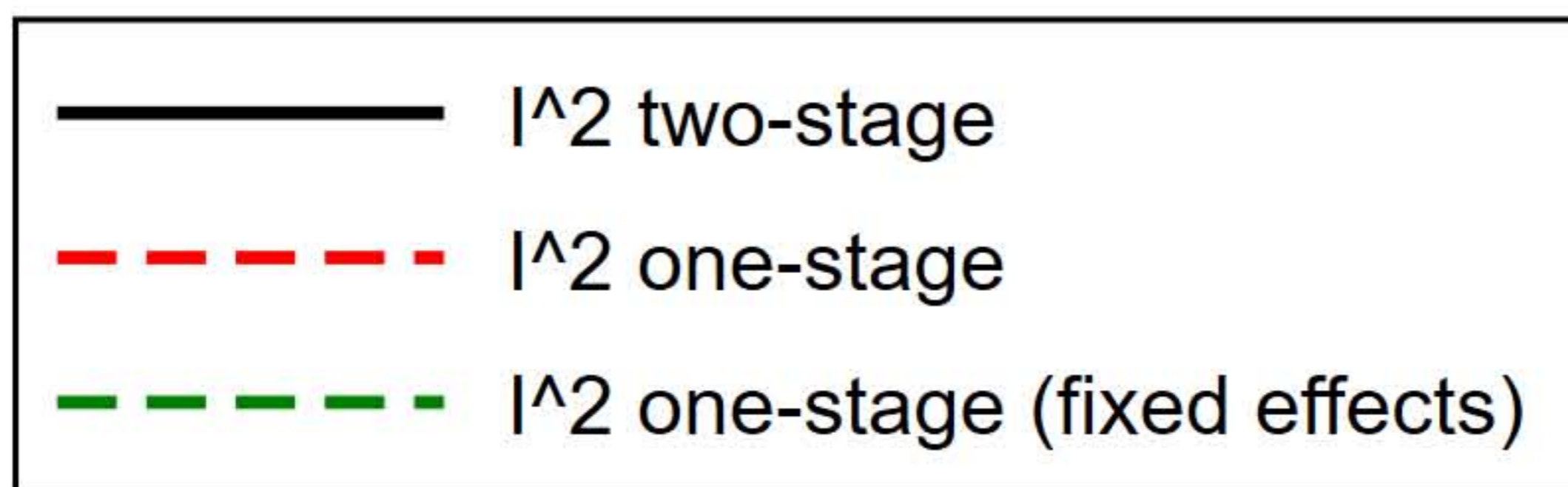

Graphs by Number per arm and Number studies per arm

Supplement: sj-pdf-1-smm-10.1177_0962280220948550 - Supplemental material for Extending the I-squared statistic to describe treatment effect heterogeneity in cluster, multi-centre randomized trials and individual patient data meta-analysis [file sj-pdf-1-smm-10.1177_0962280220948550.pdf]

10 studies

50 studies

100 studies

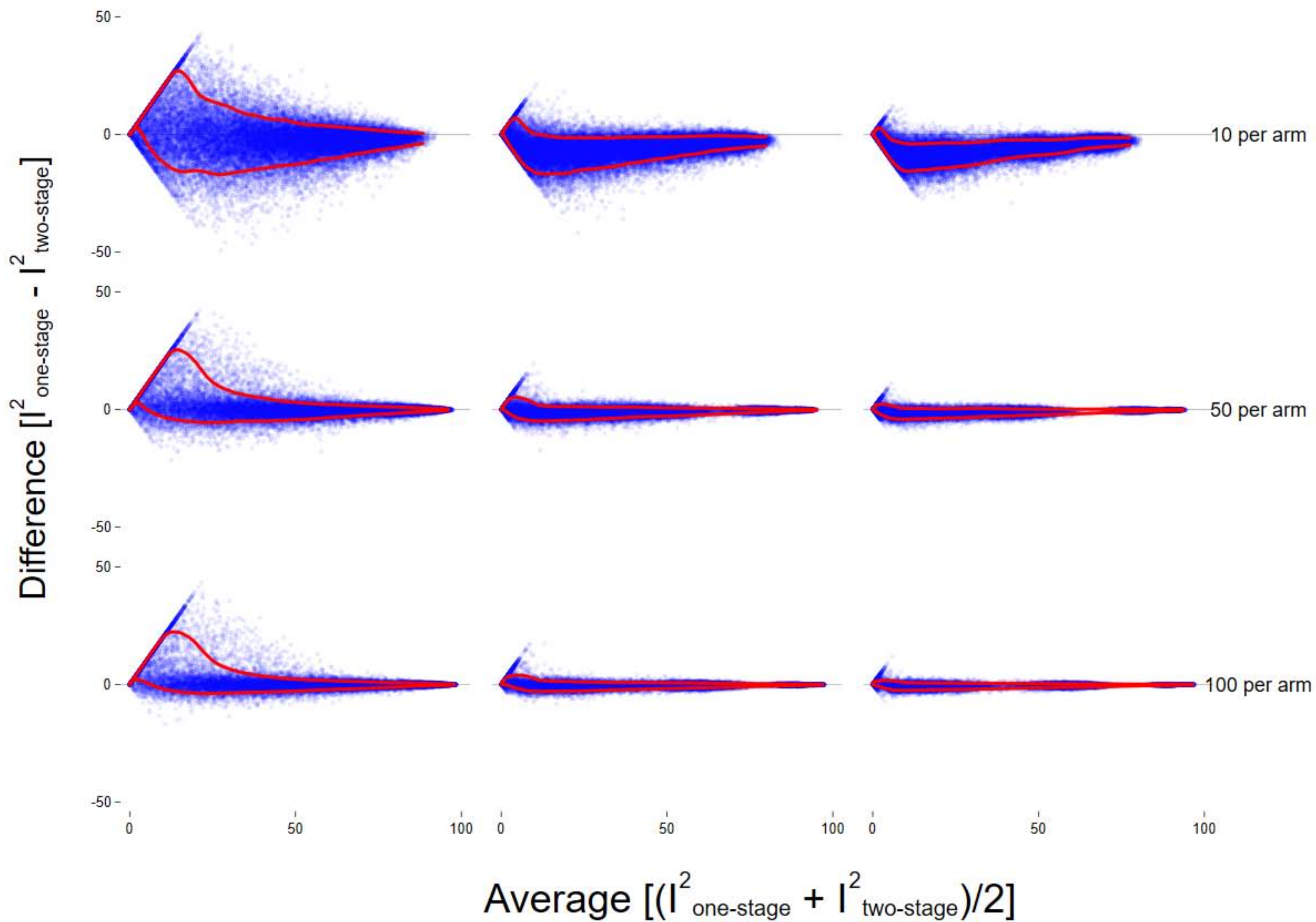

Supplement: sj-pdf-2-smm-10.1177_0962280220948550 - Supplemental material for Extending the I-squared statistic to describe treatment effect heterogeneity in cluster, multi-centre randomized trials and individual patient data meta-analysis [file sj-pdf-2-smm-10.1177_0962280220948550.pdf]

Study

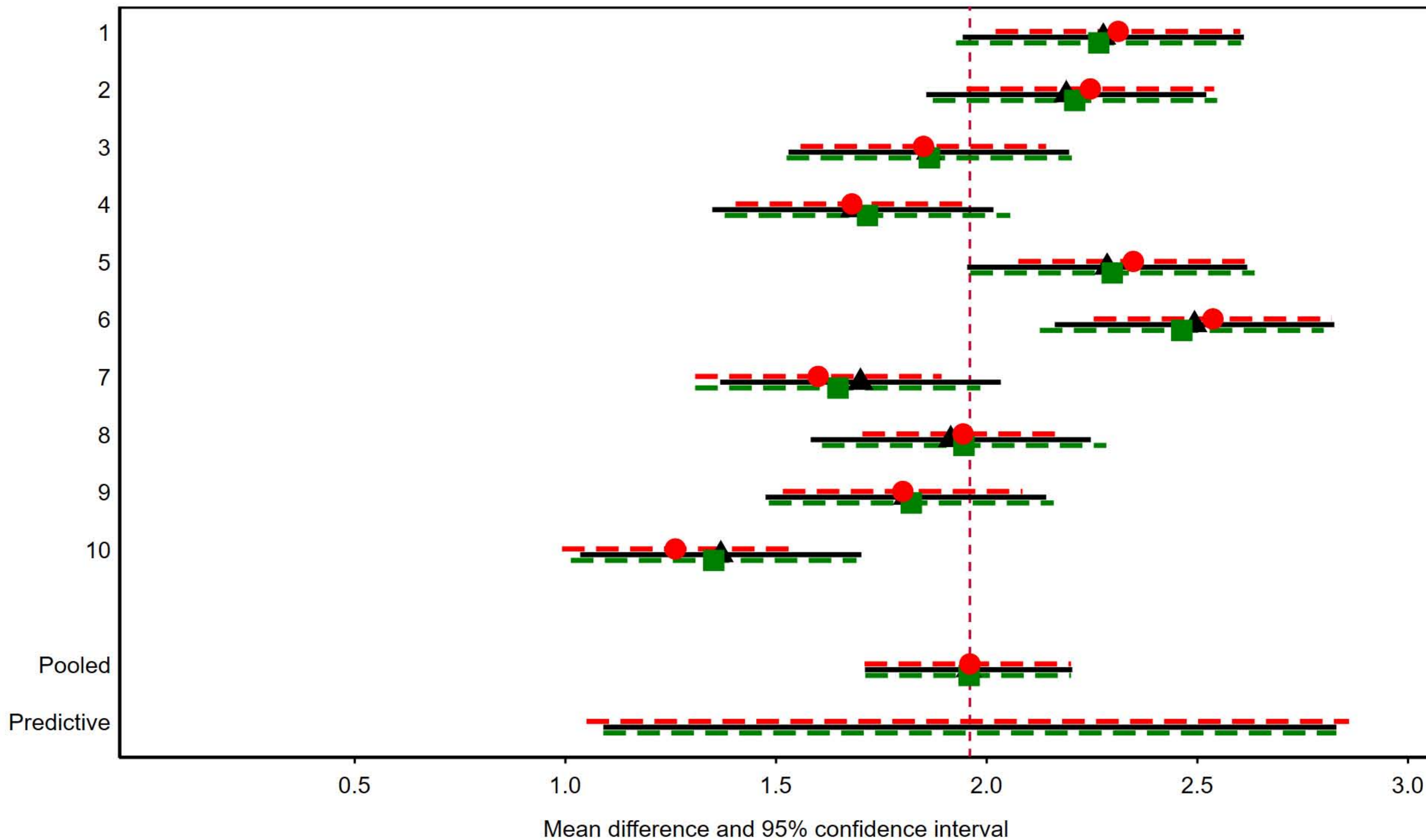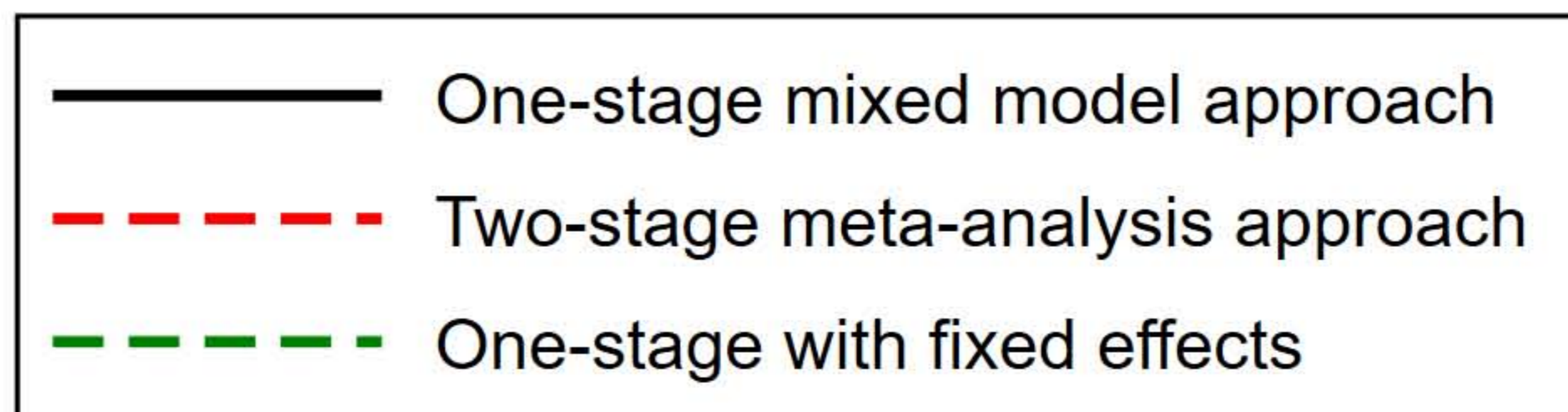

Supplement: sj-pdf-3-smm-10.1177_0962280220948550 - Supplemental material for Extending the I-squared statistic to describe treatment effect heterogeneity in cluster, multi-centre randomized trials and individual patient data meta-analysis [file sj-pdf-3-smm-10.1177_0962280220948550.pdf]
